# Susceptibility of germinating seedlings of European and Eurasian populations of *Pinus sylvestris* to damping‐off caused by *Fusarium circinatum*

**DOI:** 10.1111/efp.12749

**Published:** 2022-06-10

**Authors:** Steve Woodward, J. Asdrubel Flores‐Pacheco, E. Jordán Muñoz‐Adalia, Pablo Martínez‐Álvarez, Jorge Martín‐García, Julio J. Diez

**Affiliations:** ^1^ School of Biological Sciences University of Aberdeen, Cruickshank Building Aberdeen Scotland, UK; ^2^ Sustainable Forest Management Research Institute (iuFOR) University of Valladolid ‐ INIA, Avenida de Madrid 44 Palencia Spain; ^3^ Facultad de Recursos Naturales y Medio Ambiente, Bluefields Indian & Caribbean University‐ BICU, Apartado postal N° 88 Avenida Universitaria Bluefields Nicaragua; ^4^ Department of Plant Production and Forest Resources University of Valladolid. Avenida de Madrid 44 Palencia Spain

**Keywords:** pathogenicity, pine pitch canker, seedlings resistance, survival analyses

## Abstract

The effect of inoculation with *Fusarium circinatum* on survival of seed and seedlings of 19 populations of *Pinus sylvestris* was examined under environmentally controlled conditions, with four treatments (0, 50, 10^3^, 10^6^ spores ml^−1^). A single seed source of *P. radiata* was included as a positive control. Germination (emergence of the plumule above the compost) and health of seedlings was assessed daily, for 85 days. Spore density had a significant effect on germination: at 50 spores ml^−1^, only germination of a Northeast Scotland population was reduced. Treatment with 1000 spores ml^−1^, however, reduced germination of six populations of *P. sylvestris* and of *P. radiata.*

Survival of emerged seedlings also varied with inoculum dose. Approximately 75% of seedlings survived 85 days after germination after inoculation with 50 spores ml^−1^. Seedlings of all populations were killed within 12–16 days of germination by the 10^3^ and 10^6^ spores ml^−1^ treatments.

Emerged seedlings of the Austrian populations showed the highest susceptibility to *F. circinatum* following treatment with 50 spores ml^−1^, although 15% of seedlings of one Austrian population (AU3) survived to the end of the experiment (85 days after germination). There was no clear pattern in survival rates of the *P. sylvestris* seedlings from other populations treated with 1000 or 1 million spores ml^−1^ due to death of all emerged seedlings within a short period.

Variations in susceptibility of different populations of *P. sylvestris* to *F. circinatum* may be used in future selection and breeding programmes to reduce the impact of the pathogen as it spreads over wider areas in Europe and Eurasia.

## 1 INTRODUCTION

Pine pitch canker (PPC) is a serious disease affecting species in the genus *Pinus* and *Pseudotsuga menziesii* (Mirb.) caused by *Fusarium circinatum* Nirenberg and O'Donnell (Nirenberg and O'Donnell, **1998**). In adult trees the most common symptom of pitch canker is a bleeding, resinous canker on the main stem, terminals or large branches. Cankers on the main stem are lethal when the stem is girdled (Hepting and Roth, **1946**; Barrows‐Broaddus and Dwinell, **1985**; Barrows‐Broaddus, **1990**). The pathogen also causes damping‐off, shoot die back and death of seedlings in nurseries (Barnard and Blakeslee, **1980**).

*Fusarium circinatum* was first detected in 1945 infecting *Pinus virginiana* Mill. in the southeastern USA (Hepting and Roth, **1946**). Since then, it has spread into many pine growing areas of the world; currently, it is present in Haiti (Hepting and Roth, **1953**), Japan (Kobayashi and Muramoto, **1989**), South Africa (Viljoen et al., **1994**; Coutinho et al., **2007**), Mexico (Guerra‐Santos and Cibrián‐Tovar, **1998**), Chile (Wingfield et al., **2002**), Korea (Cho and Shin, **2004**), Uruguay (Alonso and Bettucci, **2009**), Colombia (Steenkamp et al., **2012**) and, more recently, Brazil (Pfenning et al., **2014**). In Europe, *F. circinatum* has been recorded in Spain (Dwinell, **1999**; Landeras et al., **2005**), France (EPPO, **2006**), Italy (Carlucci et al., **2007**) and Portugal (Bragança et al., **2009**). The European and Mediterranean Plant Protection Organization (EPPO) currently includes *F. circinatum* in the A2 list (present in the EPPO region but not widely distributed) of organisms recommended for regulation as quarantine pathogens.

There are at least 60 species of *Pinus,* along with *Pseudotsuga menziesii*, known to be susceptible to *F. circinatum* (Bezos et al., **2017**; Drenkhan et al. **2020**); in addition, other genera in the Pinaceae have proved susceptible in artificial inoculations (Martínez‐Álvarez et al., **2014**; Martín‐García et al., **2017**; Martín‐García et al., **2018**). However, susceptibility within the genus *Pinus* varies significantly, with *Pinus radiata* considered the most susceptible species (Wingfield et al., **2008**).

*Pinus sylvestris* (Scots pine) is the most widespread pine species in nature, covering over 28 million hectares in Europe (Stanners et al., **1995**); it is also one of the most commercially important pines and delivers a range of ecosystem services (Houston Durrant et al., **2016**). Many provenances of Scots pine have been delineated and within these provenances, further sub‐populations (hereafter ‘populations’) have been distinguished, based on a range of phenotypic characters (Salmella et al. **2013**). Scots pine is affected by many different pests and diseases (e.g. *Ips* spp., *Heterobasidion* spp.); however, potential introductions of alien pathogens with which the host has not coevolved are major concerns (Stenlid and Oliva, **2016**). For example, several reports have indicated the susceptibility of Spanish, Czech and Polish provenances of *P. sylvestris* to *F. circinatum* (Pérez‐Sierra et al., **2007**; Iturritxa et al., **2012**, **2013**, Martínez‐Alvarez et al., 2014, **2016**; Martín‐García et al., **2018**; Davidenko et al., **2018**).

Variations in susceptibility to pitch canker have also been found at an intraspecific level among provenances of *Pinus* and *Pseudotsuga* in work conducted in Brazil, Colombia, El Salvador, Guatemala, Mexico, South Africa, USA (Hodge and Dvorak, **2000**, **2007**; Gordon et al., **2006**; Dvorak et al., **2009**; Mitchell et al., **2012**; Steenkamp et al., **2012**). In Europe, most work to date has been on screening variations in susceptibility of *P. pinaster* (Vivas et al., **2012**, **2013**; Elvira‐Recuenco et al., **2014**).

Climatic conditions are considered as limiting for spread of *F. circinatum* in Northern and Central/Eastern Europe (Möykkynen et al., **2015**). However, the potential distribution of pitch canker in Europe was expected to include the Netherlands and Denmark, based on climatic change scenarios used in modelling (Watt et al., **2011**; Möykkynen et al., **2015**); climate change could, in addition, render pines in parts of the British Isles, susceptible to the disease. Climatic limitations are less of a constraint to *F. circinatum* causing damping‐off in forest nurseries, however, particularly where plants are raised under protection, and any international trade in live plant material further amplifies the high risk of the pathogen spreading to disease‐free regions of Europe where Scots pine plantations and native forests occur.

In the present work, it was hypothesized that high genetic variability in European and Eurasian Scot pine populations (Belletti et al., **2012**; Donnelly et al., **2016**; Wójkiewicz et al., **2016**) will result in differences in susceptibility of these populations to *F. circinatum*. The aim of the work reported here was to determine variations in susceptibility to pitch canker of germinating seed and young plants of nineteen Scots pine populations from several European and Eurasian provenances.

## 2 MATERIALS AND METHODS

### 2.1 Fungal material

Isolate FcCa6 of *F. circinatum* was selected for this work as it was used in other pathogenicity studies (Martínez‐Alvarez et al., **2012**, **2014**; Cerqueira et al., **2017**). The fungus was maintained on potato dextrose agar (PDA; Oxoid) at 25°C. Spore suspensions were produced by transferring five agar plugs (5 mm^2^) plus mycelium to 250 ml Erlenmeyer flasks containing 50 ml potato dextrose broth. Flasks were incubated on an orbital shaker at 180 rpm for 24 h. Spores were recovered by passing cultures through two layers of cheesecloth to remove mycelial fragments. Spore density was adjusted to 50, 10^3^ or 10^6^ spores ml^−1^ using replicate haemocytometer counts.

### 2.2 Inoculations of plant materials

A total of 19 populations of *Pinus sylvestris* and one of *Pinus radiata* (used as a positive control) were tested against *F. circinatum* in the inoculation assay (Table 1). Seventy six seeds per population were sown in cell trays (cell volume 96 ml), with one seed per cell, in a substrate of peat ‐ vermiculite 1:1 (v/v), previously autoclaved twice at 120°C for 20 min. Four different treatments were tested for each population: three spore concentrations (50, 10^3^, 10^6^ spore ml^−1^) plus the control treatment. After sowing the seeds, each cell was inoculated with 100 μl of the respective spore suspension or with sterile distilled water. Trays were maintained at 25°C with a photoperiod of 16/8 h of light/darkness and watered periodically with sterile distilled water.

**TABLE 1:** Populations of *Pinus sylvestris* and *Pinus radiata* inoculated with *Fusarium circinatum*, coded and sorted by country of origin, giving geographic coordinates. Germination rate given is based on negative control treatments

Code	Seed source	Country	Species	Coordinates		Germination % ± SE
				North	West	
AU1	Hochwolkersdorf	Austria	*Pinus sylvestris*	47° 39′ 37″	16° 16′ 56”	78.95 ± 0.13
AU2	Burgeralpe	Austria	*P. sylvestris*	47° 46′ 45”	15° 19′ 36”	52.63 ± 0.01
AU3	Fronsburg	Austria	*P. sylvestris*	48° 48′ 10”	15° 18′ 38”	68.42 ± 0.14
AU4	Tyrol	Austria	*P. sylvestris*	15° 07′ 00”	48° 11′ 00”	63.16 ± 0.23
AB	Abernethy ‐ East Central	United Kingdom	*P. sylvestris*	56° 19′ 57″	03° 18′ 44″	73.68 ± 0.16
BA	North East	United Kingdom	*P. sylvestris*	55° 00′ 00″	01° 52′ 00″	78.95 ± 0.11
BE	Beinn Eighe ‐ North West	United Kingdom	*P. sylvestris*	57° 35′ 37″	05° 25′ 45″	89.47 ± 0.01
CCC	Coille Coire Chuilc ‐ South Central	United Kingdom	*P. sylvestris*	56° 23′ 27″	04° 44′ 30″	63.16 ± 0.22
GA	North Central ‐ Glen Affric	United Kingdom	*P. sylvestris*	57° 17′ 00”	45° 56′ 00”	84.21 ± 0.00
GE	North Glen Eiwig	United Kingdom	*P. sylvestris*	55° 57′ 00”	03° 12′ 00”	68.42 ± 0.17
GL	Glen Loy ‐ South West	United Kingdom	*P. sylvestris*	56° 55′ 00”	05° 07′ 60”	78.95 ± 0.18
GR1	Drama Region	Greece	*P. sylvestris*	41° 29′ 49”	24° 26′ 19”	68.42 ± 0.12
PO1	Bytów	Poland	*P. sylvestris*	54° 08′ 00″	17° 30′ 00″	100.00 ± 0.09
PO2	Krucz	Poland	*P. sylvestris*	50° 41′ 16”	16° 00′ 44”	78.95 ± 0.01
PO3	Woziwoda	Poland	*P. sylvestris*	53° 41′ 00”	17° 57′ 00”	89.47 ± 0.10
SE2	Tornik	Serbia	*P. sylvestris*	44° 11′ 26”	19° 31′ 26”	84.21 ± 0.24
TU1	Gatacik‐Degirmendere	Turkey	*P. sylvestris*	39° 58′ 20”	31° 07′ 18”	78.95 ± 0.16
TU2	Gatacik‐Gumelidere	Turkey	*P. sylvestris*	39° 58′ 20”	31° 07′ 18”	100.00 ± 0.20
SP	Valsaín, Segovia	Spain	*P. sylvestris*	40° 53′ 51”	04° 00′ 17”	73.68 ± 0.15
RAD	Sierra de Guadarrama	Spain	*Pinus radiata*	40° 47′ 00″	03° 59′ 00″	84.21 ± 0.15

Seed germination was evaluated daily, recording the number of living and dead seedlings. At the end of the experiment, *F. circinatum* was re‐isolated from the seedlings, processing needles, stems and roots separately. Each plant part was immersed in water for 3 min, followed by 3% sodium hypochlorite for 2 min, and finally 70% alcohol for 2 min. Subsequently, tissues were rinsed in sterile distilled water for 5 min before plating on PDA amended with streptomycin sulphate (0.5 g/L) in 90 mm Petri dishes and incubating at 25°C in dark for 7 days. Fungal isolates were identified morphologically (Leslie and Summerell, **2006**).

### 2.3 Statistical analysis

Chi‐square tests (*χ*2) were applied to determine whether *F. circinatum* caused pre‐emergence mortality equally on the Scots pine populations at the three inoculum doses tested (50, 10^3^, 10^6^ spore ml^−1^). Yates' correction for continuity was applied in those cases where the expected frequencies were below 5. Survival analysis based on the Kaplan–Meier nonparametric estimator (Kaplan and Meier, **1958**) was carried out with the ‘Survival’ package (Therneau, **2017**) to test post‐emergence mortality to the end of the experiment. Survival curves were created with the ‘Survfit’ function and differences between the curves tested with the ‘Survdiff’ function. All analyses were performed using R software environment (R Foundation for Statistical Computing,).

## 3 RESULTS

Germination of control seedlings of *P. sylvestris* differed significantly between provenances (*p* > 0.05). The Polish provenances PO1, PO2 and PO3 had germination rates of ca. 95%, 77% and 84%, respectively. In contrast, three Austrian provenances (AU2, AU3 and AU4) had lower germination rates, ranging between 26%–58%.

Germination rates were reduced in the presence of *F. circinatum*, but varied according to the inoculum dose used. At the lowest dose (50 spores ml^−1^), only germination of the North East Scotland population (BA) was affected (*χ*2 = 5.22, *p* = .02). However, in inoculations with 1000 spores ml^−1^, germination rates were significantly reduced in *P. radiata* (*χ*2 = 7.24, *p* < 0.01) and in six populations of *P. sylvestris*: two UK populations (BA; *χ*2 = 6.91, *p* < 0.01 and GA; *χ*2 = 6.76, *p* < 0.01), two from Turkey (TU1; *χ*2 = 3.89, *p* = 0.04 and TU2; *χ*2 = 4.95, *p* = 0.03), one from Austria (AU2; *χ*2 = 4.38, *p* = 0.04) and the Greek provenance (GR1; *χ*2 = 10.80, *p* < 0.01). In contrast, when 10^6^ spores ml^−1^ were inoculated, results were inconclusive; germination was reduced in three Scot pine populations out of the nineteen (GA: *χ*2 = 7.24, *p* < 0.001; PO1: *χ*2 = 7.76, *p* < 0.01; TU2: *χ*2 = 6.91, *p* < 0.01).

**FIGURE 1:**
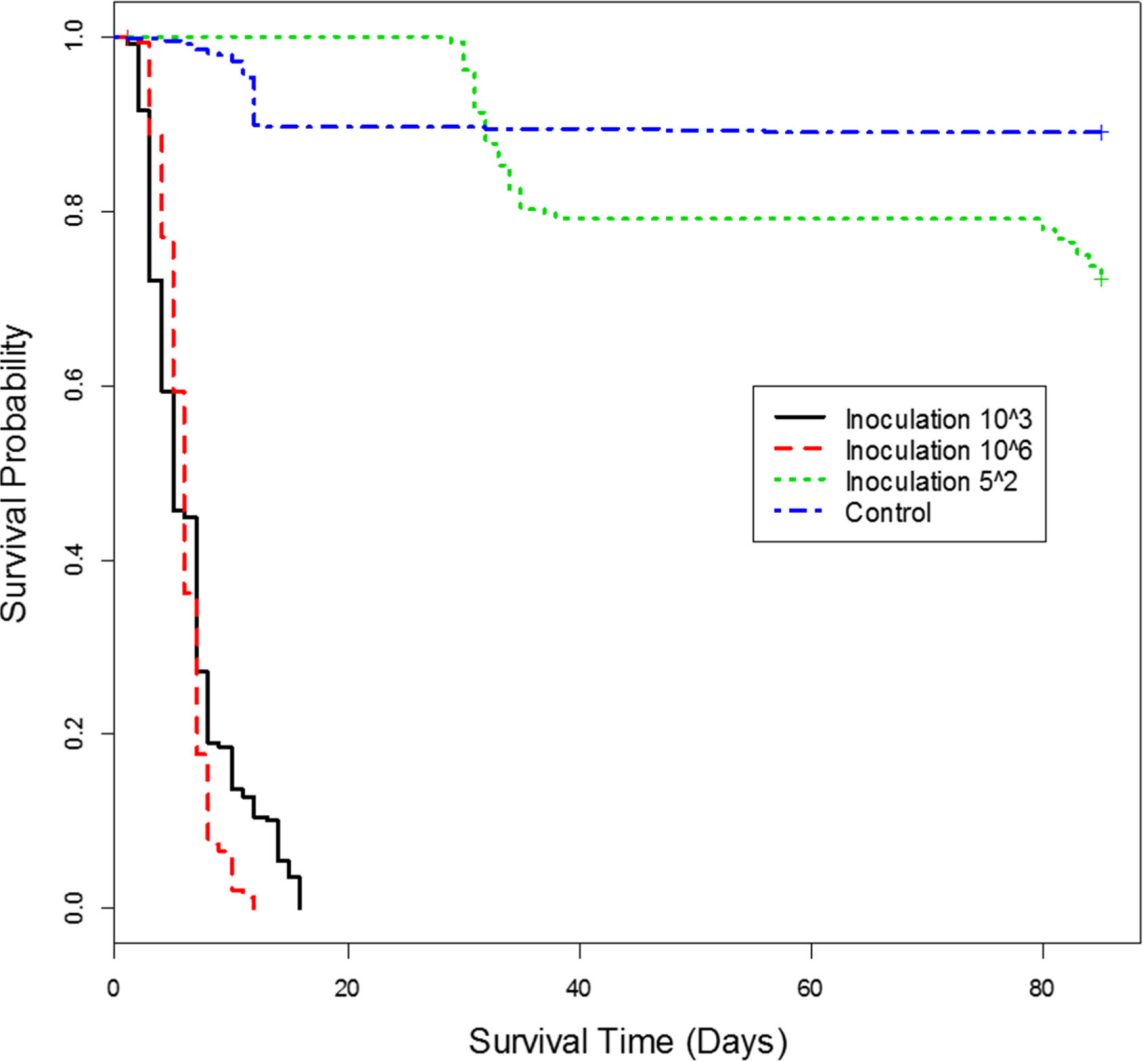
Plot of survival probability determined using the Kaplan–Meier estimate of the survival function for all populations of *Pinus sylvestris* inoculated with *fusarium circinatum* at three inoculum doses

The pathogenicity of *F. circinatum* was clearly visible in the post‐emergence mortality rates. Survival of emerged seedlings was significantly reduced by the pathogen, although it varied with inoculum dose (Figure 1; *χ*2 = 1959, *p* < 0.001). Inoculum doses of 10^3^ and 10^6^ spores ml^−1^, killed all seedlings of all populations tested by 12–16 days after germination, respectively (Figure 1). Approximately 75% of seedlings survived by 85 days after germination in the lowest inoculum dose (50 spores ml^−1^), significantly fewer than in the control treatment (*χ*2 = 33.1, *p* < 0.001).

**FIGURE 2:**
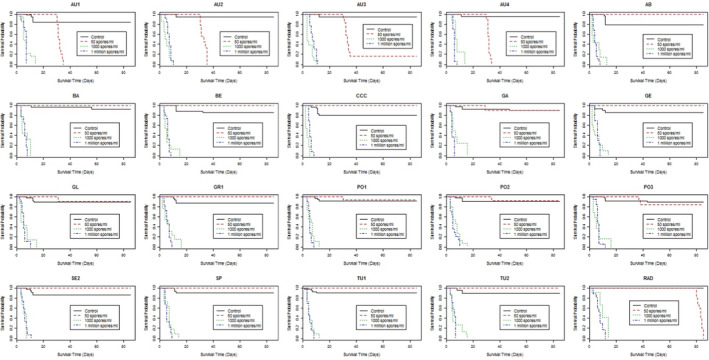
Plot of survival probability determined using the Kaplan–Meier estimate of the survival function for each population of *Pinus sylvestris* inoculated with *fusarium circinatum* at three inoculum doses. *Pinus radiata* (RAD) was similarly inoculated as a positive control. See Table 1 for population codes

**FIGURE 3:**
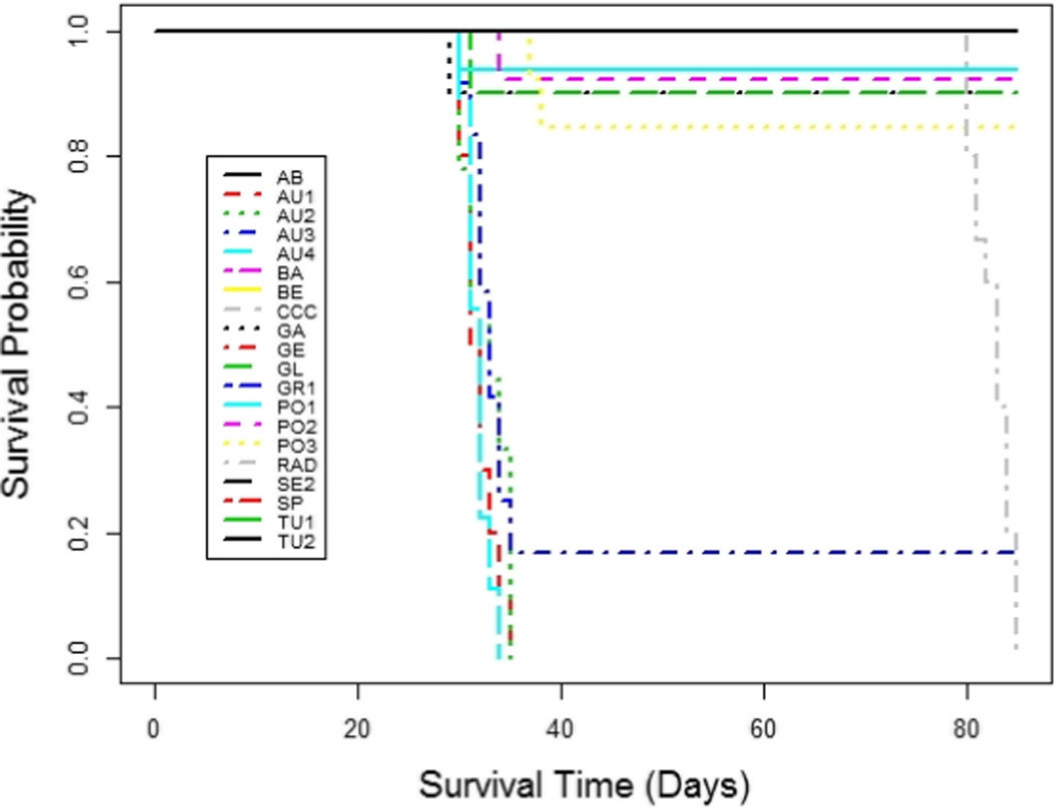
Plot of survival probability determined using the Kaplan–Meier estimate of the survival function for each population of *Pinus sylvestris* inoculated with 50 spores ml^−1^ *fusarium circinatum*. See Table 1 for population codes

**FIGURE 4:**
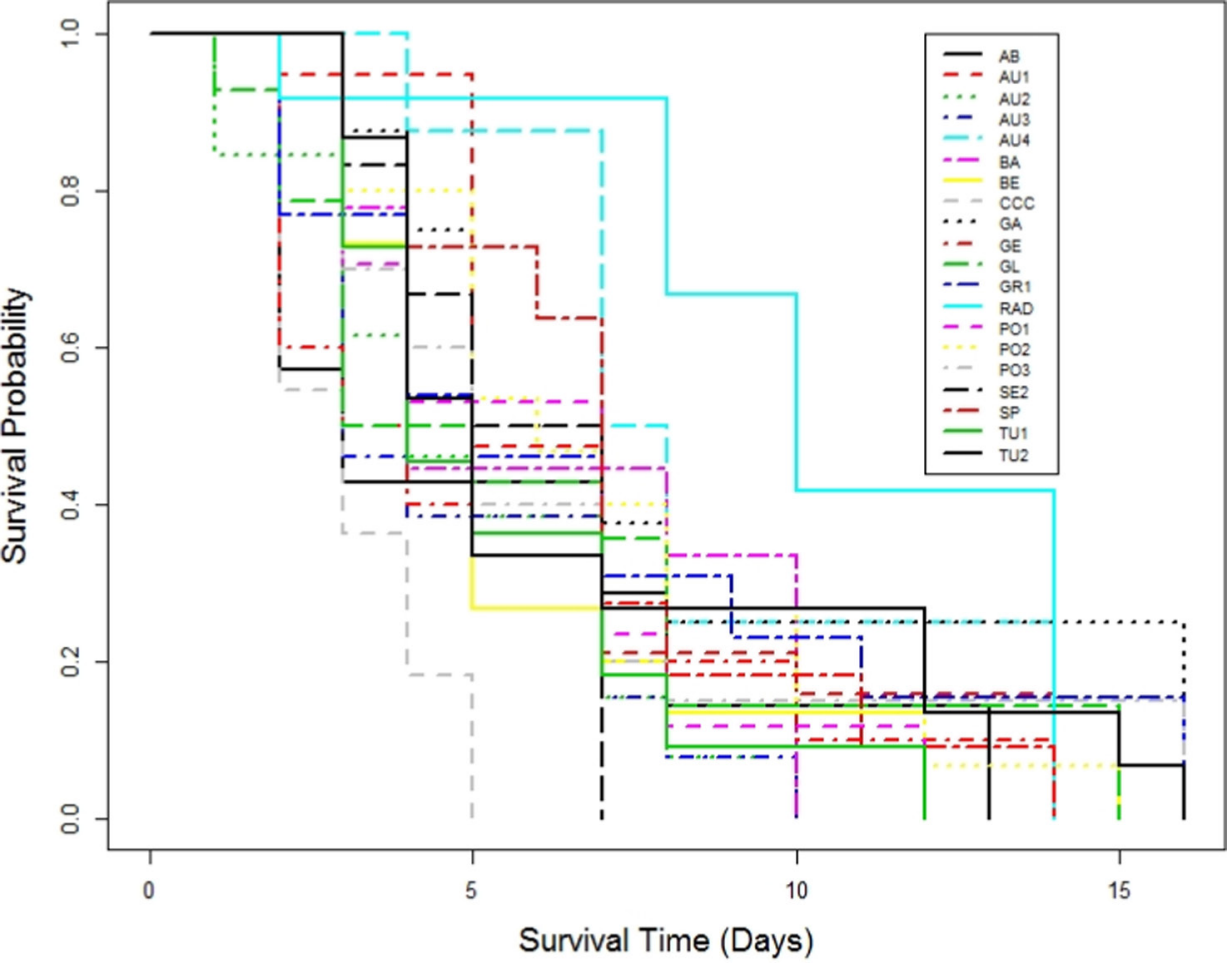
Plot of survival probability determined using the Kaplan–Meier estimate of the survival function for each population of *Pinus sylvestris* inoculated with 1000 spores ml^−1^ *fusarium circinatum*. See Table 1 for population codes

**FIGURE 5:**
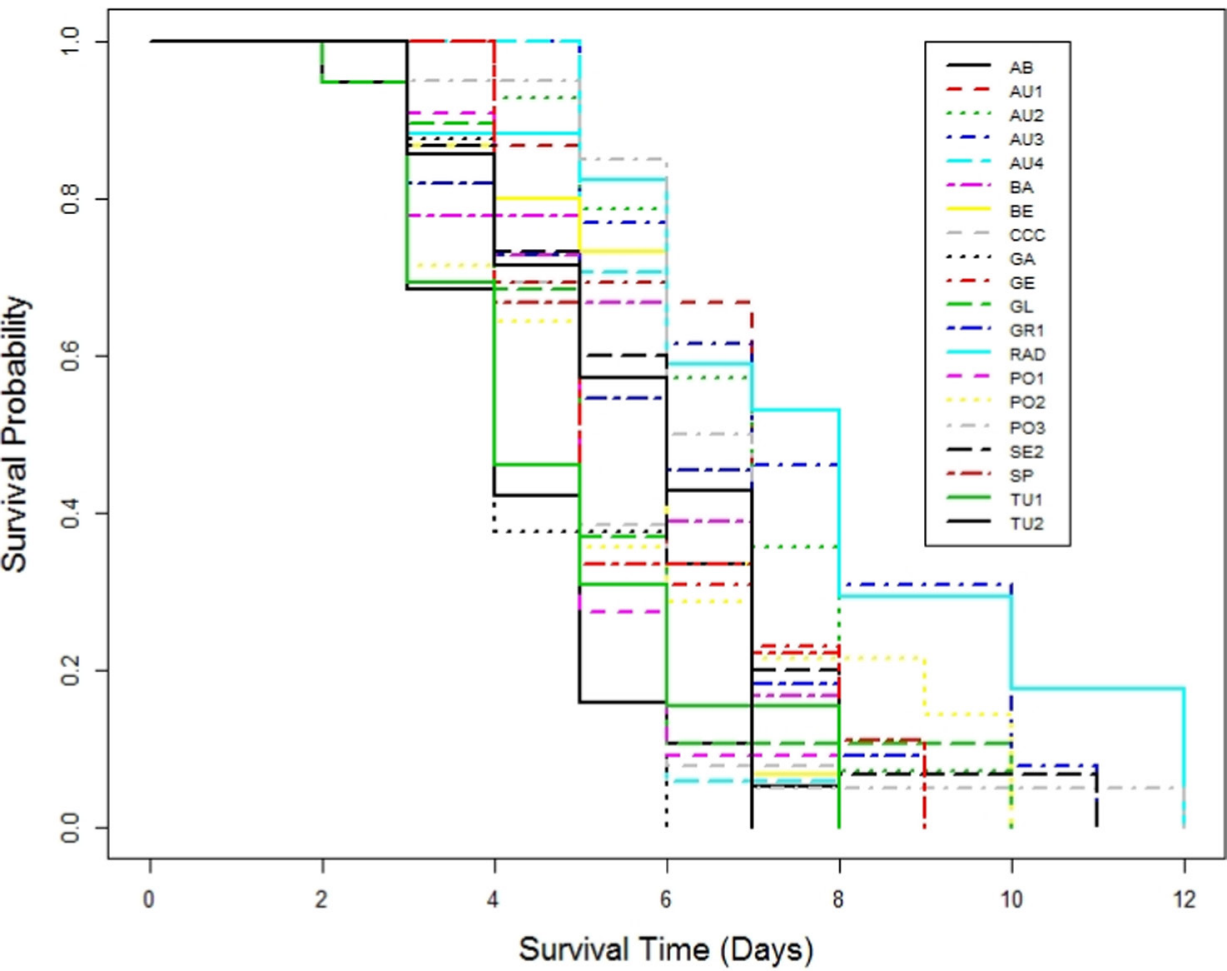
Plot of survival probability determined using the Kaplan–Meier estimate of the survival function for each population of *Pinus sylvestris* inoculated with 10^6^ spores ml^−1^ *fusarium circinatum*. See Table 1 for population codes

Differences among populations were found at each inoculum dose. At the lowest inoculum dose (50 spores ml^−1^), only Austrian provenances showed high susceptibility to *F. circinatum* (Figures 2 and 3). Mortality of *P. sylvestris* in the Austrian provenances was greater at 35 days after germination than in *P. radiata* seedlings at 80 days after germination. All seedlings of provenances AU1, AU2 and AU4 died within 35 days of germination, but ca. 15% of AU3 seedlings survived to the end of the experiment (85 days after germination). However, no significant differences in time to mortality were found among these Austrian provenances (*χ*2 = 5.1, *p* = 0.17). Although survival analyses on seedlings treated with 1000 or 1 million spores ml^−1^ showed significant differences among populations (*χ*2 = 42.8, *p* < 0.001 and *χ*2 = 60.2, *p* < 0.001, respectively), no clear pattern in survival was found due to death of all emerged seedlings within a short period (Figures 4 and 5).

## 4 DISCUSSION

The work described in this paper demonstrated that provenances and sub‐populations of Scots pine from different regions of Europe and northern Turkey vary in susceptibility to damping‐off following inoculation *Fusarium circinatum*. Nursery contamination with *F. circinatum* is probably the main mechanism for subsequent escape of the pathogen into plantations (e.g. Santana et al., **2016**), although the diseases caused by the same pathogen in the two environments differ in aetiology. Previous work has shown that young Scots pine plants from more restricted ranges in Europe vary in response to *F. circinatum* (Pérez‐Sierra et al., **2007**; Iturritxa et al., **2012**, **2013**; Martín García et al., **2017**, **2018**; Davydenko et al., **2018**), but this work was the first to show responses in germinating seed and non‐suberized seedlings, based on the examination of 17 different European and two Turkish provenances/populations of Scots pine to *F. circinatum*; responses of a single provenance of *P. radiata* were also tested, as a positive control.

Currently, PPC disease in Europe is restricted to areas where *P. radiata* is commonly used in forest plantations. Under the climatic conditions in Northern Spain, where *F. circinatum* was probably introduced in the 1990s (Dwinell, **1999**; Landeras et al., **2005**), the disease is causing substantial damage to the highly susceptible *P. radiata*. Other pine species are at risk, however, since many species in the genus and native to or grown in Europe and Eurasia are known to be susceptible to *F. circinatum* (Iturritxa et al., **2012**, **2013**, Martínez‐Alvarez et al., **2014**, **2016**); moreover, changing climate is likely to lead to an expansion in the area suitable for establishment of the pathogen (Watt et al., **2011**; Möykkynen et al., **2015**). Among the pine species at risk to this disease in Europe, *P. sylvestris* is arguably the most important because of its wide distribution from western Europe to the far east of Russia, although the high genetic variability within the species (Wójkiewicz et al., **2016**) may mitigate against *F. circinatum* infection of different provenances and sub‐populations in natural stands.

Several reports have described the relative susceptibility of different *Pinus* species to *F. circinatum* (McCain et al., **1987**; Gordon et al., **1998**, **2006**, Iturritxa et al., **2012**, **2013**, Martínez‐Alvarez et al., **2014**, **2016**). Against other pathogens, however, variations in susceptibility are known to be higher among provenances of the same species than between different species, as reported for *Gremmniella abietina* (Lagerb.) M. Morelet attacking *P. sylvestris*, *Pinus contorta* Dougl. and *Picea abies* (L.) H. Karst (Hansson, **1998**).

Differences in germination were found among populations tested in this work. Variations in in germination rates of *P. sylvestris* seeds between populations observed here have also been reported previously for other populations of this species (Reich et al., **1994**; Tilki, **2005**; Martínez‐Alvarez et al., **2012**). Factors other than the origin of the seeds, such as their age or the storage conditions used, may also influence germination rates. When inoculated with *F. circinatum*, a small reduction in apparent germination, based on appearance of the plumule above soil level occurred, varying with inoculum dose. Similar reductions in the germination of several pine species, including *P. sylvestris*, *P. radiata*, *P. pinaster*, *P. nigra*, *P. strobus* and *P. uncinata* were reported previously (Martínez‐Alvarez et al., **2014**), using a single inoculum dose rate of 10^6^ spores ml^−1^; no analysis of the impacts of spore density was performed in the earlier work.

The pathogen had a much greater impact on post‐emergence survival of seedlings than on germination rates. The two highest spore concentrations of the pathogen applied killed almost all the seedlings of all populations. Although when compared with higher inoculum doses, the pathogen was less damaging when applied at 50 spores ml^−1^, approximately 25% of resulting seedlings died at this low dose rate. It is important to emphasize that at a total spore dose of 5 spores per plant, *F. circinatum* was able to reduce the germination of the North East Scotland pine population (BA) and kill all seedlings of three Austrian provenances (AU1, AU2 and AU4), together with all plants of *P. radiata*, illustrating the potential of this pathogen for destroying pine plants in contaminated forest nurseries. Other authors reported lesions developing on seedlings of several pine species, after inoculating 25 or 50 spores (Gordon et al., **1998**; Iturritxa et al., **2012**, **2013**), but to our knowledge symptoms have never been reported previously when using such a low number of spores.

The unsuberized tissues of newly germinated seedlings used in this work could have favoured colonization by the pathogen, ultimately contributing to the high rates of mortality. In contrast, the two higher doses of inoculum applied in the work, 10^3^ and 10^6^ spores ml^−1^ resulted in rapid death of all seedlings, with no clear discernible pattern in susceptibility among populations/provenances. In other experiments performed with 10^6^ spores ml^−1^, a similar rapid death of pine seedlings also occurred (Martínez‐Alvarez et al., **2014**): fewer than 10% of *P. sylvestris* seedlings survived after inoculation. In contrast, whereas two Romanian provenances of *Pinus mugo* and *Picea abies* were clearly susceptible to *F. circinatum* at the lowest inoculum dose tested (50 spores ml^−1^), a *P. sylvestris* provenance was not susceptible at any dose (Martín‐García et al., **2017**). Mortality was higher in *P. radiata* inoculated in both the present work, and in that reported by Martínez‐Alvarez et al. (**2012**), compared with *P. sylvestris*. As mentioned above, *P. radiata* appears to be the pine species most susceptible to *F. circinatum*, and a high mortality rate is expected in this host compared with most other *Pinus* spp. However, when 50 spores ml^−1^ were applied, the Austrian provenances tested in the present work, AU1, AU2 and AU4, had the same mortality rate as *P. radiata,* of 100%. In contrast, with higher spore concentrations, it took over 80 days for the last *P. radiata* seedling to die, whereas seedlings of the three Austrian provenances of *P. sylvestris* died within 35 days of inoculation. The remaining Austrian provenance, AU3 was also very susceptible to the disease, although, despite most seedlings of this provenance dying within 35 days of inoculation, approximately 15% survived until the end of the experiment. Further inoculation experiments with these Austrian provenances are required to explore the reasons for the high susceptibility to *F. circinatum*.

This work confirmed that the selection of a provenance is an important consideration when planning forest plantations in the presence of pathogen threats. It is generally recommended that, when feasible, the provenance native to the region in which the plantations will be established is the preferred option, as it is adapted to local conditions. In the altered conditions presented by the establishment of an alien invasive pathogen in a given region; however, exploring potential variations in resistance between provenances of a tree species should be considered. It is important, therefore, to conduct screening for relative resistance/susceptibility between provenances of pines so that appropriate forest management decisions can be made when *F. circinatum* spreads to and establishes in other regions of Europe.

## Data Availability

Raw data are available to legitimate users on request from the corresponding author.
